# Do occupational health and safety tools that utilize artificial intelligence have a measurable impact on worker injury or illness? Findings from a systematic review

**DOI:** 10.1186/s13643-025-02869-1

**Published:** 2025-07-11

**Authors:** Arif Jetha, Hela Bakhtari, Emma Irvin, Aviroop Biswas, Maxwell J. Smith, Cameron Mustard, Victoria H. Arrandale, Jack T. Dennerlein, Peter M. Smith

**Affiliations:** 1 Institute for Work & Health, Toronto, ON Canada; 2 Dalla Lana School of Public Health, University of Toronto, Toronto, ON Canada; 3 School of Health Studies, Faculty of Health Sciences, Western University, London, ON Canada; 4 Seargent College of Health and Rehabilitation Sciences, Boston University, Boston, MA USA

**Keywords:** Artificial intelligence, Occupational health and safety practice, Injury prevention, Technological adoption

## Abstract

**Background:**

Artificial intelligence (AI) holds promise as a tool that can be used by practitioners in the field of occupational health and safety (OHS). This study aimed to identify AI applications specifically used for OHS and examine their impact on worker morbidity or mortality outcomes.

**Methods:**

We conducted a comprehensive systematic review. We searched six databases to identify published quantitative studies of OHS AI applications across the hierarchy of controls that were published between years 2018 to 2024. Title/abstract and full-text screening was conducted to identify eligible studies which were then assessed for quality and risk of bias and synthesized.

**Results:**

Of the 1255 articles identified by our search, only two met eligibility criteria; one of which was appraised as medium quality and the other as low quality. The one medium quality study identified by our review was an AI-based chatbot health promotion tool which was shown to improve musculoskeletal symptoms. Our systematic review shows that we are at the early stages of understanding the role AI can play in OHS and it may be premature to recommend the wide-spread use of AI for health and safety practice within workplaces.

**Conclusion:**

There is a critical need for future research to unpack how considerations taken in the development and adoption of workplace AI tools for OHS can determine their effectiveness in addressing worker injury or illness.

**Systematic review registration:**

PROSPERO CRD42023414422.

**Supplementary Information:**

The online version contains supplementary material available at 10.1186/s13643-025-02869-1.

## Background

Artificial intelligence (AI) holds promise as a tool that can be used by practitioners in the field of occupational health and safety (OHS) within workplaces to prevent worker morbidity or mortality [1, 2]. The ability to process large amounts of diverse data to generate predictions and automate a range of functions means that AI has the potential to advance our ability to rapidly identify and address the most complex OHS challenges [3–5]. While enthusiasm regarding the role of AI within the labour market is high, there remains limited information on whether the technology can have a marked impact on worker health and safety outcomes. An emerging body of literature in the field of OHS suggests that there could be potential risks and benefits of the technology for OHS, yet empirical evidence and outcome data for its use in this context remains minimal [1, 2].

Currently, research has focused on the role of AI as an analytical tool that can be applied to big datasets to produce estimates of the risk and severity of occupational injury or illness and identify underlying patterns in data [6–9]. It is unclear to what extent tools exist within workplaces that leverage AI and can be used to identify and eliminate diverse hazards, manage and support OHS decisions or promote health and safety behaviours. It is also unclear whether the adoption of OHS AI applications can contribute to a reduction of worker injuries, illnesses, or fatalities.

Within industrialized labour market contexts, the burden of occupational morbidity and mortality on workers, workplaces and society is substantial [10]. According to data from the International Labour Organization (ILO), on a global scale, occupational accidents and diseases are estimated to account for up to 2.78 million fatalities each year while non-fatal work-related injuries and illnesses impact an additional 374 million workers [11]. Despite ongoing improvements in OHS policy and practice, the rate of occupational injury or illness resulting in a lost-time claim has remained stable [12–14]. What is more, the OHS landscape is becoming increasingly complex [10]. The growing acknowledgement of diverse physical and psychosocial workplace hazards related to a range of occupational injuries such as mental health stress [15], physical and psychological injuries stemming from workplace violence [16] or COVID-19 exposure [17] have meant that workplaces and OHS professionals are facing more challenges in protecting workers from occupational injuries and illness [18]. Of concern, recent data also show that in some industrialized jurisdictions, the rate of workplace fatalities have plateaued or even increased over the last decade [13, 19, 20]. In Canada, 1056 workers died from a work-related cause in 2023, reflecting an increase in workplace fatalities in recent years [21]. Indeed, there is a need to continue to innovate prevention practices to address the burden of occupational injury.

Leveraging AI represents an opportunity to improve the prevention of occupational injury and illness and eliminate workplace fatalities [1]. AI is an umbrella term broadly referring to the use of computing machines to solve problems traditionally requiring human intelligence, including detecting patterns, making predictions and decisions, or optimizing processes [3]. At the heart of AI are algorithms (sequences of mathematical operations) that are trained using large labelled or unlabeled datasets. Under the AI umbrella is machine learning (ML) which involves the application of statistical algorithms to large amounts of data with the aim of making probabilistic inferences about a range of outcomes [22]. ML is being adopted by workplaces to detect patterns in structured or unstructured data, make predictions, generate decisions, or optimize processes [5, 23]. Deep learning, a subset of ML that uses neural networks (i.e. algorithms with structures inspired by human brain function), has driven recent advancements in image recognition and natural language processing [24–26].

Growing affordability, improved computational power, and the increased availability of big data have meant that AI is more accessible and has widespread applicability to different functions within workplaces [4]. There are examples of AI applications across diverse sectors which have shown to increase efficacy and productivitiy in job tasks including healthcare (e.g. diagnostic medical imaging [27]), financial services (e.g. automated fraud detection tools [28]) and transportation (e.g. real-time identification of obstacles in automated vehicles [29]). In many of these cases, AI is integrated into existing technology to enhance its capabilities to make predictions and automate different aspects of work (e.g. smart sensors, robots, mobile technologies, Internet of Things devices [1]). There currently is a limited understanding of the extent to which AI is used in OHS practice within workplaces.

Emerging technologies have often been utilized by OHS professionals including sensing and warning technologies to alert workers of occupational hazards, wearables to detect unsafe ergonomic practices and digital management platforms to coordinate organizational OHS decision-making. Data have shown that the adoption of these technologies have improved organizational OHS practices and have contributed to a reduction in the prevalence of worker injuries or illnesses [30–34]. It is important to highlight that past technologies have been designed to perform discrete functions which are dictated by predefined conditions and may be limited in their ability to respond and adapt to complex and dynamic health and safety challenges.

The ability to learn sets AI apart from past OHS technological applications and enables the technology to make more rapid and accurate predictions when confronting new health and safety threats. As a result, workplace AI applications have the potential to automate a greater range of OHS activities with less human control. Within the context of smarter work environments where workers in diverse industries are closely connected with digital technologies, AI applications can be used in conjunction with other advancing digital technologies to monitor physical, psychosocial, cognitive, and biomechanical workplace conditions to enhance the detection of workplace hazards and implement more timely hazard control measures [30]. At the same time, limitations in the scope of parameters included in the algorithms which underpin AI and the selection of datasets used to train AI that can lack sufficient size, representation or diversity may be sources of bias that can contribute to an unequal distribution of benefits of the technology and may limit its effectiveness within the field [2, 35].

Through a systematic review of literature, our study aims to better understand the role of OHS AI applications in workplace health and safety and its impact on worker morbidity and mortality. Study objectives are to:

1. Identify and categorize workplace applications that use AI for addressing and enhancing OHS practice.
2. Examine and explain how the design and adoption of OHS AI applications can contribute to changes in worker injury, illness or fatalities.

## Methods

We conducted a systematic review of published literature using a process developed by the Cochrane Collaboration and was adapted by the Institute for Work & Health (IWH) Systematic Review Program [36]. The review methods were registered with PROSPERO (CRD42023414422) and meet the 2020 Preferred Reporting Items for Systematic Reviews and Meta-analyses (PRISMA) statement guidelines. A populated PRISMA checklist can be viewed in more detail (see Additional file 1). The systematic review process was also informed by engagement with health and safety stakeholders whose insights informed the search and synthesis approaches and interpretation of key findings [2].

### Literature search

The search strategy followed a Population (P), Exposure (E), Comparator (C) and Outcome (O) (PECO) framework to capture studies that examined the impact of workplace OHS tools that explicitly harnessed AI technology. We searched for studies of workers in high- and middle-income industrialized economies including those in the Organisation of Economic Cooperation and Development (OECD) as well as China, India, Brazil, Russia (P) who were exposed to any specific workplace OHS application or tool where the integration of AI was explicitly mentioned and its impact on worker injury, illness or fatalities were described (E). To note, our search focused on OHS AI applications across the hierarchy of controls including those that aimed at identifying and eliminating hazards, managing and supporting OHS decisions or promoting health and safety behaviours. Our search included studies with any comparison group (C) and that measured any worker occupational morbidity or mortality outcomes including mental or physical injuries, incidence of occupational illness or disease or workplace fatalities (O) (Table 1).

**Table 1:** Inclusion and exclusion criteria using PECO framework

PECO category	Inclusion criteria	Exclusion criteria
Population	• Any workers in high- and middle-income industrialized economies including those in the OECD as well as China, India, Brazil or Russia	• Workers in other labour market contexts
Exposure	• Exposure to any specific workplace occupational health and safety (OHS) application or tool where integration of AI was explicitly mentioned • Any workplace AI OHS application across the hierarchy of controls	• OHS applications where the use of AI was not explicitly mentioned
Comparison	• Any comparison group	• No comparison exists
Outcome	• Any occupational injury • Any occupational illness or disease • Any occupational fatality	• Occupational injury, illness, disease or fatality data not presented

Database-specific controlled vocabulary terms and keywords were included and are available in Supplement 1, Additional file 2. The terms within each category were combined using a Boolean OR operator and terms across the four main categories were combined using a Boolean AND operator. Embase (OVID), PsycINFO (OVID), Sociological Abstracts, ASSIA, ABI Inform were searched for articles that were published between January 2018 and April 2024. Our decision to focus on articles published from 2018 onwards was made to reflect the more recent prominence of AI-related applications within workplaces. After removing duplicates, the search yields were combined and imported into the review software DistillerSR to facilitate relevance screening [37]. Our search was conducted in May 2023 and updated January 2024.

### Relevance screen

Relevance screening occurred over two steps. First, titles and abstracts of references identified in the search were divided among research team members and were screened independently by two reviewers for relevancy. Any disagreements were resolved by a third reviewer. Articles that were relevant at the first level of screening were carried forward for a full-text review, which was carried out by two reviewers. Disagreements between the two reviewers were discussed in team meetings. Relevance decisions on title and abstract and full-text screening demonstrated moderate to high inter-rater reliability suggesting that reviewers were consistently applying the inclusion/exclusion criteria to screening processes. Reference lists of eligible articles were checked to ensure no additional relevant articles were missed.

Abstract and full-text relevancy screening was informed by our PECO framework. Articles were included if they were primary research, published $\ge$ 2018, focused on workers in middle- or high-income industrialized economies, focused on a specific AI OHS application and assessed any worker occupational morbidity or mortality outcome. We identified only quantitative study designs where statistical effect estimates were reported and that enabled us to attribute changes in outcomes to the AI application and could potentially enable us to pool findings for an evidence synthesis. Accordingly, studies were excluded if they were secondary research, qualitative research, gray literature, commentaries, editorials or case studies. Studies were excluded if AI was used for analytical purposes to examine an OHS topic using existing data rather than a specific AI tool adopted within a workplace. Also, studies were excluded if they described prototypes or piloted an AI OHS application and did not collect information on worker morbidity and mortality.

### Quality appraisal and data extraction

A modified version of a quality assessment tool developed by IWH for systematic reviews in the field of OHS was used to conduct quality appraisal [38]. The quality assessment tool consisted of 23 questions that examined internal, external and statistical validity of each article through an assessment of study design and objectives, recruitment procedures, outcome and exposure measurement and analysis. Questions were added to the quality appraisal tool to examine the extent to which the AI was described including the parameters of the algorithm or a description of the training data used to inform the AI (see Supplement 2, Additional file 2).

Each relevant article was appraised by two independent reviewers. A final weighted sum score of the quality criteria was generated and converted to a percentage score. Using the percentage score, studies were categorized as high (≥ 85%), medium (50–84%) or low quality (< 50%). Research team members held meetings to reach consensus on final appraisal scores and rankings. Only studies appraised as high and medium quality were utilized in the evidence synthesis phase. Data were extracted from relevant articles to create summary tables that described the study sample, AI tool and its impact on OHS outcomes (see Tables 2 and 3). Data synthesis aimed at examining how AI applications were designed (e.g. algorithm parameters and use of training data) and how they functioned to mitigate hazards and improve OHS outcomes (Tables 1 and 2).

**Table 2:** Description of studies identified in our systematic review of evidence

**Author, year and country**	**Study objectives**	**Study design**	**Measurement period**	**Sample size and description**	**Occupations **	**Type of AI tool **	**Injury or illness type**	**Outcome measures **
**Anan T et al., 2021, Japan**	To evaluate the improvements in musculoskeletal symptoms in workers with neck/shoulder stiffness/pain and low back pain after the use of an exercise-based artificial intelligence (AI)–assisted interactive health promotion system that operates through a mobile messaging application	Two-armed randomized control trial	12 weeks	Intervention group: • *N*=48; 19% women Control group • *N*=46; 28% women	• Engineers • Clerical workers	Chatbot AI-assisted prevention program that was available on mobile applications	• Musculoskeletal disorders	• Subjective stiffness and pain assessments of the neck and shoulder • Low back pain • Subjective improvements of pain over study period

**Table 3:** Description of occupational health and safety artificial intelligence tool and key study findings

**Author, Year**	**Description of AI tool**	**Description of control group treatment**	**Findings **
**Anan T, et al., 2021**	• The AI-assisted mobile chatbot was programed to send users messages with exercise instructions and some tips on what they can do in their daily lives to improve those symptoms. • Messages were sent every day through a mobile application which prompted the user to perform exercises that could be finished within 1 minute, and included stretching, maintaining good posture, and mindfulness. • The program was interactive and users could ask questions to the chatbot and receive tailored replies. • The chatbot could also determine when the participant’s exercise was interrupted and motivate them to continue.	• Regular exercise routine, which included exercising for about 3 minutes during the break time provided by the company every day to prevent stiff shoulders and back pain.	• At 12 weeks, neck/shoulder stiffness/pain or low back pain in the intervention group (mean = 3.0, SD 1.1) was significantly lower than the control group (mean = 4.0, SD 0.8) (P<.001). • In the intervention group, the proportion of participants who had severe symptoms decreased from 77% at baseline to 33% (12 weeks). The proportion of participants with severe symptoms in the control group decreased from 76% to 67%. • 75% of participants reported subjective improvement in pain in the intervention group when compared to 7% of participants in the control group. • The intervention group was significantly less likely to report a difference in the worst pain scores of neck/shoulder pain/stiffness and low back pain between baseline and 12 weeks when compared to the control group (OR = -1.12; 95% CI –1.5, –0.70) • Participants in the intervention group showed significant improvements in neck/shoulder pain/stiffness and low back pain severity compared to the control group (OR= 6.36, 95% CI, 2.57-15.73). • The intervention group was significantly more likely to report a subjective improvement in symptoms (OR = 43.00 (95% CI 11.25-164.28).

### Evidence synthesis

The limited number of eligible studies identified by the review coupled with variability in the observation length, intervention type, sample characteristics and outcomes meant that we were unable to calculate pooled effect estimates. Instead, a narrative synthesis is used to describe the findings.

## Results

**Fig. 1:**
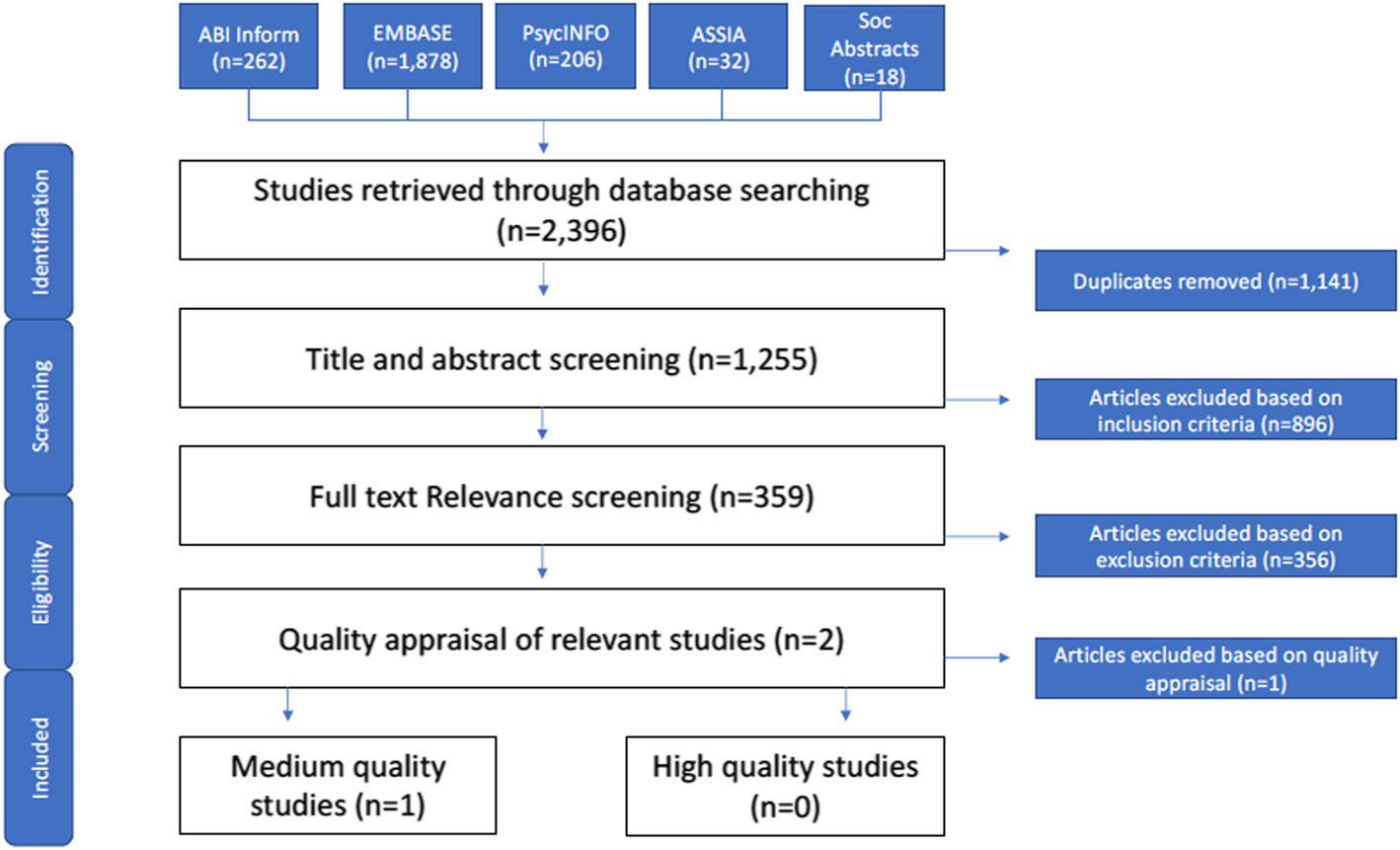
Systematic review search and synthesis overview

Our search yielded 1255 articles that were published between 2018 and 2024 after duplicates were removed and met initial eligibility criteria. Through title and abstract screening, 359 articles met initial selection criteria and were carried forward for full-text review. A total of two studies were found to meet eligibility criteria. Using the quality appraisal tool, no studies were of high quality. One article was of medium quality and one article was appraised as being of low quality (see Fig. 1).

Among studies that were excluded during full-text reivew, 10% focused on a workplace AI OHS application within an industrialized labour market context but did not examine its impact on worker morbidity or mortality outcomes.

The one study that was uncovered was a randomized control trial study design conducted by Anan et al. [39] which tested the effect of an AI-based chatbot health promotion system on musculoskeletal symptoms (MSK). This study was conducted among 94 white collar workers in Japan. The chatbot provided health promotion instructions to participants that included exercise instructions and specific suggestions to improve musculoskeletal symptoms including shoulder neck pain and stiffness and low back pain. Findings indicated that the intervention group exhibited significant improvements in neck and shoulder pain and stiffness and low back pain severity compared to control group (OR = 6.36, 95% CI, 2.57–15.73). The intervention group was also significantly more likely to report a subjective improvement in symptoms (OR = 43.00, 95% CI 11.25–164.28).

## Discussion

The working world is undergoing an AI revolution which is characterized by an increasing use of intelligent machines to automate workplace processes and job tasks across a range of industries and occupations. Our study revealed that we are still in the early phases of understanding the extent to which AI can enhance workplace health and safety practices. Only a single study was identified by our systemic review which examined the impact of OHS AI applications on occupational morbidity and mortality. In the absence of evidence, recommendations regarding the use of AI for OHS cannot be made. Findings underscore the importance of ongoing primary research to evaluate how the design and application of AI for OHS purposes can impact worker health and safety.

We utilized an established and rigorous systematic review methodology designed for studies in the field of OHS to search and synthesize available peer-reviewed research [36]. Our systematic review approach focused on identifying workplace OHS applications which leverage AI and examine the effects of technological adoption on worker morbidity and mortality. We identified one medium quality study. The single study was an AI chatbot health promotion application to prevent worker musculoskeletal disorders [39]. The automated and tailored recommendations provided by the AI chatbot were effective in reducing musculoskeletal symptoms among the intervention group. Given the single study, we were unable to determine whether AI chatbots or other similar forms of AI applications could be effective in addressing occupational injuries, illnesses or fatalities. Indeed, our systematic review suggests that it may be early to recommend the wide-spread use of AI for health and safety practice within workplaces. We also have a limited understanding of the workplace conditions or worker-level personal or health factors that can determine whether an OHS AI tool is effective.

While there is a growing discourse on the benefits of AI applications and tools on all aspects of working life, our study offers important findings by showing that we know very little about the utility of AI for OHS. The absence of literature on the topic is indeed a significant finding and speaks to the need for research on the topic. Our review provides a foundation for future research focusing on OHS AI tools implemented in the workplace and the importance of measuring impact.

Future research will benefit from a transdisciplinary approach by drawing on subject matter experts from diverse disciplines (e.g. computer science, OHS, social policy), using a variety of methodologies, data collection approaches and analytical tools to advance an evidence base that unpacks the complexities of AI use in the workplace and its impacts on OHS and worker health outcomes [40–42]. Given the infancy of this body of literature, research on the impact of AI tools should collect information on a range of OHS outcome measures including workplace injuries, occupational disease and workplace fatalities. In addition, there may be a need to develop new quality appraisal tools for research on the impact of AI on worker health and safety within the workplace which could be particularly useful as the evidence base grows. It is important to acknowledge that our study only focused on published peer-reviewed quantitative evidence. Also, our study may not have captured OHS AI tools developed by private firms whose design details may be proprietary and may not have published findings regarding the efficacy of the tool for public critique. Moreover, our systematic review excluded pilot studies of OHS AI prototypes. As described in the results, close to 10% of articles uncovered in our review were feasibility studies that were conducted within lab-based settings. For example, The pilot studies identified in our review found that AI-enabled robotic systems within industrial and health care settings could be effective in preventing worker injuries [43, 44]. However, these studies were not included in our final synthesis as they offered limited generalizability to real-world workplace settings and their impact on worker morbidity and mortality could not be ascertained. As these pilot tools are implemented and tested, additional research is needed to examine their effectiveness. Future evidence synthesis approaches that summarize other forms of evidence (e.g. qualitative research, gray literature, blogs, commercial websites) may enhance our understanding of different AI tools being used within workplaces for OHS and how they may impact workers.

AI’s capacity to learn, adapt and create outputs with increasing independence means that it could hold promise as a tool to address the most complex workplace health and safety challenges [3, 4, 24, 45]. Our study was not able to examine how considerations in the design, training or implementation of AI tools could contribute to its effectiveness or distribution of benefits for different groups of workers. Growing numbers of technologists and multidisciplinary scholars have called for the design of responsible AI where AI systems are developed, assessed and implemented in safe, trustworthy and ethical ways that are free from bias and do not harm different groups of workers [46]. Algorithms that are designed with parameters that are limited in scope or the training of AI with data that may lack diversity or representativeness can be critical sources of bias that contribute to AI underperforming across different groups of workers and an unequal distribution of benefits and burdens of the technology [2, 35, 47]. Examining and addressing bias in the design of AI OHS applications can ensure that the technology does not cause unanticipated harms to different groups of workers and inform strategies that safeguard those who are exposed to the technology [48, 49]. We show that there is a paucity of research which have explored how AI design considerations may impact the performance of the technology to address OHS outcomes and the extent to which the technology can be beneficial or harmful for different groups of workers. What is more, increasing research highlights that ongoing data requirements can be a source of concern for workers who may fear loss of privacy and constant surveillance [50, 51]. Accordingly, the implementation of AI may have unintended consequences for worker health and safety. In sum, there is a need for future research to unpack how considerations taken in the development and adoption of workplace AI tools can determine its effectiveness in addressing morbidity and mortality and may be used to guide responsible and safe AI design.

Growing data availability and increased computing power are doubling the operating speed of AI every 4 to 9 months, resulting in an evolving learning capacity of AI and a changing impact on workers and workplaces [26, 52]. It is expected that increasingly independent and autonomous forms of AI are on the horizon. For instance, at the time of this article, innovations in deep learning were at the forefront of machines being able to match or surpass humans in performing certain types of tasks, including some involving image and speech recognition. The impact and use of AI for OHS purposes will grow over time and necessitate a research approach to monitor and understand emerging AI applications used in OHS and their positive and negative impact on worker health and safety. One way to study the constant change to AI and its impact on OHS practice may be through living systematic review and evidence approaches [53]. Living systematic reviews involve regularly and systematically surveilling and synthesizing evidence to generate an up-to-date understanding of AI innovation and usage as it relates to the health and safety of workers.

## Conclusions

Despite advancements and proliferation of AI within the working world, our understanding of how the technology may actually benefit OHS practice remains very limited. Our comprehensive systematic review of peer-reviewed literature identified only one study which tested the impact of an AI OHS application on the morbidity of workers. We are still in the early phases of an understanding of how the technology may be used as a tool for OHS practitioners to address the health and safety of workers. Indeed, ongoing research is needed to monitor and track the efficacy of new workplace OHS AI applications and their impact on different occupational injuries and illnesses. Importantly, future research on the impact of AI adoption on the field of OHS will enable strategic responses that ensure that AI is being adopted for health and safety purposes in ways that address risks and maximize its benefits.

## Supplementary Information

Additional file 1: File format: Microsoft Word DOC. Title of data: PRISMA 2020 Checklist. Description of data: Populated PRISMA Checklist for this review.

Additional file 2: File format: Microsoft Word DOC. Title of data: Supplement 1: Database specific search terms and Supplement 2: Quality appraisal criteria and question weight. Description of data: Supplement 1 provides the database specific (i.e., Embase (OVID), PsycINFO (OVID) Sociological Abstracts, ASSIA, ABI) search terms that were used for this review. Supplement 2 provides the Quality appraisal criteria and associated weighting that were applied to each relevant article identified through the search and screening process.

## Data Availability

None.

## Abbreviations

AI: Artificial intelligence
OHS: Occupational health and safety
