# Supplementary material for: Do occupational health and safety tools that utilize artificial intelligence have a measurable impact on worker injury or illness? Findings from a systematic review
Source: Syst Rev. 2025 Jul 11;14:146. doi: 10.1186/s13643-025-02869-1 (PMC12247322; doi:10.1186/s13643-025-02869-1)
Supplement: Supplementary file 2 — Additional file 2: File format: Microsoft Word DOC. Title of data: Supplement 1: Database specific search terms and Supplement 2: Quality appraisal criteria and question weight. Description of data: Supplement 1 provides the database specific (i.e., Embase (OVID), PsycINFO (OVID) Sociological Abstracts, ASSIA, ABI) search terms that were used for this review. Supplement 2 provides the Quality appraisal criteria and associated weighting that were applied to each relevant article identified through the search and screening process. [file 13643_2025_2869_MOESM2_ESM.docx]

**Supplement 1: Database specific search terms**

| *Database 1: Embase (N=1031)*  Database: Embase Classic+Embase <1947 to 2023 October 11>  Search Strategy:  --------------------------------------------------------------------------------  1 (Worker? or labo?rer?).ti,ab.  2 employee?.ti,ab.  3 employer?.ti,ab.  4 employment/  5 employment.ti,ab.  6 job?.ti,ab.  7 occupation*.ti,ab.  8 work/  9 workplace/  10 worksite*.ti,ab.  11 "work site*".ti,ab.  12 industry.ti,ab.  13 ("labour market" or "labor market").ti,ab.  14 or/1-13  15 artificial intelligence/  16 "artificial intelligence".ti,ab.  17 machine learning/  18 "machine learning".ti,ab.  19 "intelligen* algorithm".ti,ab  20 "collective intelligence".ti,ab.  21 "prediction machine".ti,ab.  22 "computer heuristic".ti,ab.  23 "expert system".ti,ab.  24 expert system/  25 "fuzzy logic".ti,ab.  26 fuzzy logic/  27 "deep learning".ti,ab.  28 deep learning/  29 "human-machine".ti,ab.  30 man machine interaction/  31 "natural language processing".ti,ab.  32 natural language processing/  33 NLP.ti,ab.  34 neural network.ti,ab.  35 artificial neural network/  36 robotic.ti,ab.  37 robotics/  38 smart sensor$.ti,ab.  39 automated reasoning.ti,ab.  40 automated reasoning/  41 computer vision.ti,ab.  42 computer vision/  43 smart machine$.ti,ab.  44 deep analytics.ti,ab.  45 big data.ti,ab.  46 decision support system$.ti,ab.  47 data mining.ti,ab.  48 data mining/  49 multi-agent systems.ti,ab.  50 health informatics.ti,ab.  51 medical informatics/  52 belief state.ti,ab.  53 online agent.ti,ab.  54 learning agent.ti,ab.  55 "strong AI".ti,ab.  56 "weak AI".ti,ab.  57 training data.ti,ab.  58 predictive analytics.ti,ab.  59 cognitive automation.ti,ab.  60 intelligent automation.ti,ab.  61 semantic analysis.ti,ab.  62 cognitive computing.ti,ab.  63 AI bias.ti,ab.  64 (AI adj3 (judgment or judgement)).ti,ab.  65 (AI adj3 prediction).ti,ab.  66 automation bias.ti,ab.  67 cloud computing.ti,ab.  68 deepfake.ti,ab.  69 data architecture.ti,ab.  70 computational thinking.ti,ab.  71 general adversarial network*.ti,ab.  72 human machine teaming.ti,ab.  73 human AI teaming.ti,ab.  74 intelligent sensing.ti,ab.  75 object recognition.ti,ab.  76 one shot learning.ti,ab.  77 reinforcement learning.ti,ab.  78 augmented reality.ti,ab.  79 semi-supervised learning.ti,ab.  80 explainability.ti,ab.  81 "generative AI".ti,ab.  82 "large language model*".ti,ab.  83 or/15-82  84 (injur* adj2 preven*).ti,ab.  85 exp occupational accident/  86 (occupation* adj2 (accident* or disease* or hazard* or health or illness* or injur* or safety)).ti,ab.  87 exp occupational disease/  88 exp occupational health/  89 exp occupational safety/  90 (workplace adj2 (hazard* or injur* or safety)).ti,ab.  91 accident/  92 fatalit*.ti,ab.  93 or/84-92  94 exp musculoskeletal disease/  95 "musculoskeletal disease".ti,ab.  96 "musculoskeletal injur*".ti,ab.  97 carpal tunnel syndrome/  98 "carpal tunnel syndrome".ti,ab.  99 tendinitis/  100 (tendinitis or tendonitis).ti,ab.  101 neck pain/  102 "neck pain".ti,ab.  103 back pain/  104 "back pain".ti,ab.  105 shoulder pain/  106 "shoulder pain".ti,ab.  107 osteoarthritis/  108 osteoarthritis.ti,ab.  109 cumulative trauma disorder/  110 "repetitive strain injur*".ti,ab.  111 RSI.ti,ab.  112 hand-arm vibration syndrome/  113 "hand-arm vibration syndrome".ti,ab.  114 "hand injur*".ti,ab.  115 bursitis/  116 bursitis.ti,ab.  117 "wounds and injuries".ti,ab.  118 patient risk prevention.ti,ab.  119 or/94-118  120 "psychol* injur*".ti,ab.  121 psychotrauma/  122 posttraumatic stress disorder/  123 "post-traumatic stress".ti,ab.  124 (PTSD or PTSS or PTSI).ti,ab.  125 "psychol* trauma".ti,ab.  126 technostress.ti,ab.  127 (psychol* adj2 strain).ti,ab.  128 stressor$.ti,ab.  129 job strain.ti,ab.  130 (job adj2 stress*).ti,ab.  131 job stress/  132 (work adj2 stress*).ti,ab.  133 (career adj2 stress*).ti,ab.  134 (employ* adj2 stress*).ti,ab.  135 or/120-134  136 93 or 119 or 135  137 14 and 83 and 136  138 limit 137 to yr=2018-2023 |
| --- |
| *Database 2: PsycINFO (N=115)*  Database: APA PsycInfo <1806 to October Week 1 2023>  Search Strategy:  --------------------------------------------------------------------------------  1 (Worker? or labo?rer?).ti,ab.  2 employee?.ti,ab.  3 employer?.ti,ab.  4 employment status/  5 employment.ti,ab.  6 job?.ti,ab. (100084)  7 occupation*.ti,ab.  8 worksite*.ti,ab.  9 "work site*".ti,ab.  10 industry.ti,ab.  11 ("labour market" or "labor market").ti,ab.  12 or/1-11  13 artificial intelligence/  14 "artificial intelligence".ti,ab.  15 machine learning/  16 "machine learning".ti,ab.  17 "intelligen* algorithm".ti,ab.  18 "collective intelligence".ti,ab.  19 "prediction machine".ti,ab.  20 "computer heuristic".ti,ab.  21 "expert system".ti,ab.  22 expert systems/  23 "fuzzy logic".ti,ab.  24 fuzzy logic/  25 "deep learning".ti,ab.  26 deep learning/  27 "human-machine".ti,ab.  28 Human Computer Interaction/  29 "natural language processing".ti,ab.  30 natural language processing/  31 NLP.ti,ab.  32 neural network.ti,ab.  33 artificial neural networks/  34 robotic.ti,ab.  35 robotics/  36 smart sensor$.ti,ab.  37 automated reasoning.ti,ab.  38 computer vision.ti,ab.  39 smart machine$.ti,ab.  40 deep analytics.ti,ab.  41 big data.ti,ab.  42 decision support system$.ti,ab.  43 data mining.ti,ab.  44 data mining/  45 multi-agent systems.ti,ab.  46 health informatics.ti,ab.  47 medical informatics/  48 belief state.ti,ab.  49 online agent.ti,ab.  50 learning agent.ti,ab.  51 "strong AI".ti,ab.  52 "weak AI".ti,ab.  53 training data.ti,ab.  54 predictive analytics.ti,ab.  55 cognitive automation.ti,ab.  56 intelligent automation.ti,ab.  57 semantic analysis.ti,ab.  58 cognitive computing.ti,ab.  59 AI bias.ti,ab.  60 (AI adj3 (judgment or judgement)).ti,ab.  61 (AI adj3 prediction).ti,ab.  62 automation bias.ti,ab.  63 cloud computing.ti,ab.  64 deepfake.ti,ab.  65 data architecture.ti,ab.  66 computational thinking.ti,ab.  67 general adversarial network*.ti,ab.  68 human machine teaming.ti,ab.  69 human AI teaming.ti,ab.  70 intelligent sensing.ti,ab.  71 object recognition.ti,ab.  72 one shot learning.ti,ab.  73 reinforcement learning.ti,ab.  74 augmented reality.ti,ab.  75 semi-supervised learning.ti,ab.  76 explainability.ti,ab.  77 "generative AI".ti,ab.  78 "large language model*".ti,ab.  79 or/13-78  80 (injur* adj2 preven*).ti,ab.  81 exp Industrial accidents/  82 (occupation* adj2 (accident* or disease* or hazard* or health or illness* or injur* or safety)).ti,ab.  83 exp Work related illnesses/  84 exp occupational health/  85 exp occupational safety/  86 (workplace adj2 (hazard* or injur* or safety)).ti,ab.  87 accidents/  88 fatalit*.ti,ab.  89 or/80-88  90 exp musculoskeletal disorders/  91 "musculoskeletal disease".ti,ab.  92 "musculoskeletal injur*".ti,ab.  93 "carpal tunnel syndrome".ti,ab.  94 (tendinitis or tendonitis).ti,ab.  95 "neck pain".ti,ab.  96 back pain/  97 "back pain".ti,ab.  98 "shoulder pain".ti,ab.  99 arthritis/  100 osteoarthritis.ti,ab.  101 "repetitive strain injur*".ti,ab.  102 RSI.ti,ab.  103 "hand-arm vibration syndrome".ti,ab.  104 "hand injur*".ti,ab.  105 bursitis.ti,ab.  106 "wounds and injuries".ti,ab.  107 patient risk prevention.ti,ab.  108 or/90-107  109 "psychol* injur*".ti,ab.  110 posttraumatic stress disorder/  111 "post-traumatic stress".ti,ab.  112 (PTSD or PTSS or PTSI).ti,ab.  113 "psychol* trauma".ti,ab.  114 technostress.ti,ab.  115 (psychol* adj2 strain).ti,ab.  116 stressor$.ti,ab.  117 job strain.ti,ab.  118 (job adj2 stress*).ti,ab.  119 occupational stress/  120 (work adj2 stress*).ti,ab.  121 (career adj2 stress*).ti,ab.  122 (employ* adj2 stress*).ti,ab.  123 or/109-122  124 89 or 108 or 123  125 12 and 79 and 124  126 limit 125 to yr=2018-2023 |
| *Database 3: ABI Inform (N=145)*  --------------------------------------------------------------------------------  S1  (Ti,ab(worker* OR laborer* OR labourer*) OR ti,ab(employee* OR employer* OR employment OR job* OR occupation* OR worksite* OR "work site*" OR industry OR "labour market" OR "labor market") OR MAINSUBJECT.EXACT("Employment")) AND PEER(yes)  S2  (ti,ab("artificial intelligence" OR "machine learning" OR "intelligen* algorithm" OR "collective intelligence" OR "prediction machine" OR "computer heuristic" OR "expert system" OR "fuzzy logic" OR "deep learning" OR "human-machine" OR "natural language processing" OR NLP OR "neural network" OR robotic OR "smart sensor*" OR "automated reasoning" OR "computer vision" OR "smart machine*" OR "deep analytics" OR "big data" OR "decision support system*" OR "data mining" OR "multi-agent systems" OR "health informatics" OR "belief state" OR "online agent" OR "learning agent" OR "strong AI" OR "weak AI" OR "training data" OR "predictive analytics" OR "cognitive automation" OR "intelligent automation" OR "semantic analysis" OR "cognitive computing" OR "AI bias" OR (AI NEAR/3 (judgment OR judgement)) OR (AI NEAR/3 prediction) OR "automation bias" OR "cloud computing" OR deepfake OR "data architecture" OR "computational thinking" OR "general adversarial network*" OR "human machine teaming" OR "human AI teaming" OR "intelligent sensing" OR "object recognition" OR "one shot learning" OR "reinforcement learning" OR "augmented learning" OR "semi-supervised learning" OR explainability or "generative AI" OR "large language model*") OR MAINSUBJECT.EXACT("Artificial intelligence") OR MAINSUBJECT.EXACT("Machine learning") OR MAINSUBJECT.EXACT("Expert systems") OR MAINSUBJECT.EXACT("Fuzzy logic") OR MAINSUBJECT.EXACT("Deep learning") OR MAINSUBJECT.EXACT("Man machine interaction") OR MAINSUBJECT.EXACT("Natural language processing") OR MAINSUBJECT.EXACT("Robotics") OR MAINSUBJECT.EXACT("Big Data") OR MAINSUBJECT.EXACT("Decision support systems") OR MAINSUBJECT.EXACT("Data mining") OR MAINSUBJECT.EXACT("Health informatics") OR MAINSUBJECT.EXACT("Predictive analytics")) AND PEER(yes)  S3  (ti,ab((injur* NEAR/2 preven*) OR (occupation* NEAR/2 (accident* OR disease* OR hazard* OR health OR illness* OR injur* OR safety)) OR (workplace NEAR/2 (hazard* OR injur* OR safety)) OR fatalit*) OR MAINSUBJECT.EXACT("Occupational accidents") OR MAINSUBJECT.EXACT("Occupational diseases") OR MAINSUBJECT.EXACT("Occupational health") OR MAINSUBJECT.EXACT("Occupational safety") OR MAINSUBJECT.EXACT("Accidents")) AND PEER(yes)  S4  (ti,ab("musculoskeletal disease*" OR "musculoskeletal injur*" OR "carpal tunnel" OR tendonitis OR "neck pain" OR "back pain" OR "shoulder pain" OR osteoarthritis OR "repetitive strain injur*" OR RSI OR "hand-arm vibration syndrome" OR "hand injur*" OR bursitis OR "wounds and injuries" OR "patient risk prevention") OR MAINSUBJECT.EXACT("Musculoskeletal diseases") OR MAINSUBJECT.EXACT("Carpal tunnel syndrome") OR MAINSUBJECT.EXACT("Tendinitis") OR MAINSUBJECT.EXACT("Neck pain") OR MAINSUBJECT.EXACT("Back pain") OR MAINSUBJECT.EXACT("Osteoarthritis") OR MAINSUBJECT.EXACT("Repetitive motion disorders") OR MAINSUBJECT.EXACT("Bursitis")) AND PEER(yes)  S5  (ti,ab("psychol* injur*" OR "post-traumatic stress" OR PTSD OR PTSS OR PTSI OR "psychol* trauma" OR technostress OR (psychol* NEAR/2 strain) OR stressor* OR "job strain" OR (job NEAR/2 stress*) OR (work NEAR/2 stress*) OR (career NEAR/2 stress*) OR (employ* NEAR/2 stress*)) OR MAINSUBJECT.EXACT("Post traumatic stress disorder") OR MAINSUBJECT.EXACT("Occupational stress")) AND PEER(yes)  S6  [S3] OR [S4] OR [S5]  S7  [S1] AND [S2] AND [S6]  S8  ([S1] AND [S2] AND [S6]) AND pd(20180101-20231031) |
| *Database 4: Sociological Abstracts (N=9)*  --------------------------------------------------------------------------------  S1  (Ti,ab(worker* OR laborer* OR labourer*) OR ti,ab(employee* OR employer* OR employment OR job* OR occupation* OR worksite* OR "work site*" OR industry OR "labour market" OR "labor market") OR MAINSUBJECT.EXACT("Work") OR MAINSUBJECT.EXACT("Workplaces") OR MAINSUBJECT.EXACT("Employment")) AND PEER(yes)  S2  (ti,ab("artificial intelligence" OR "machine learning" OR "intelligen* algorithm" OR "collective intelligence" OR "prediction machine" OR "computer heuristic" OR "expert system" OR "fuzzy logic" OR "deep learning" OR "human-machine" OR "natural language processing" OR NLP OR "neural network" OR robotic OR "smart sensor*" OR "automated reasoning" OR "computer vision" OR "smart machine*" OR "deep analytics" OR "big data" OR "decision support system*" OR "data mining" OR "multi-agent systems" OR "health informatics" OR "belief state" OR "online agent" OR "learning agent" OR "strong AI" OR "weak AI" OR "training data" OR "predictive analytics" OR "cognitive automation" OR "intelligent automation" OR "semantic analysis" OR "cognitive coputing" OR "AI bias" OR (AI NEAR/3 (judgment OR judgement)) OR (AI NEAR/3 prediction) OR "automation bias" OR "cloud computing" OR deepfake OR "data architecture" OR "computational thinking" OR "general adversarial network*" OR "human machine teaming" OR "human AI teaming" OR "intelligent sensing" OR "object recognition" OR "one shot learning" OR "reinforcement learning" OR "augmented learning" OR "semi-supervised learning" OR explainability OR "generative AI" OR "large language model*") OR MAINSUBJECT.EXACT("Artificial Intelligence") OR MAINSUBJECT.EXACT("Expert Systems") OR MAINSUBJECT.EXACT("Worker Machine Relationship")) AND PEER(yes)  S3  (ti,ab((injur* NEAR/2 preven*) OR (occupation* NEAR/2 (accident* OR disease* OR hazard* OR health OR illness* OR injur* OR safety)) OR (workplace NEAR/2 (hazard* OR injur* OR safety)) OR fatalit*) OR MAINSUBJECT.EXACT("Occupational Safety and Health") OR MAINSUBJECT.EXACT("Accidents")) AND PEER(yes)  S4  ti,ab("musculoskeletal disease*" OR "musculoskeletal injur*" OR "carpal tunnel" OR tendonitis OR "neck pain" OR "back pain" OR "shoulder pain" OR osteoarthritis OR "repetitive strain injur*" OR RSI OR "hand-arm vibration syndrome" OR "hand injur*" OR bursitis OR "wounds and injuries" OR "patient risk prevention") AND PEER(yes)  S5  (ti,ab("psychol* injur*" OR "post-traumatic stress" OR PTSD OR PTSS OR PTSI OR "psychol* trauma" OR technostress OR (psychol* NEAR/2 strain) OR stressor* OR "job strain" OR (job NEAR/2 stress*) OR (work NEAR/2 stress*) OR (career NEAR/2 stress*) OR (employ* NEAR/2 stress*)) OR MAINSUBJECT.EXACT("Posttraumatic Stress Disorder") OR MAINSUBJECT.EXACT("Trauma") OR MAINSUBJECT.EXACT("Occupational Stress")) AND PEER(yes)  S6  [S3] OR [S4] OR [S5]  S7  [S1] AND [S2] AND [S6]  S8  ([S1] AND [S2] AND [S6]) AND pd(20180101-20231031) |
| *Database 5: ASSIA (N=18)*  --------------------------------------------------------------------------------  S1  (Ti,ab(worker* OR laborer* OR labourer*) OR ti,ab(employee* OR employer* OR employment OR job* OR occupation* OR worksite* OR "work site*" OR industry OR "labour market" OR "labor market") OR MAINSUBJECT.EXACT("Work") OR MAINSUBJECT.EXACT("Workplaces") OR MAINSUBJECT.EXACT("Employment")) AND PEER(yes)  S2  (ti,ab("artificial intelligence" OR "machine learning" OR "intelligen* algorithm" OR "collective intelligence" OR "prediction machine" OR "computer heuristic" OR "expert system" OR "fuzzy logic" OR "deep learning" OR "human-machine" OR "natural language processing" OR NLP OR "neural network" OR robotic OR "smart sensor*" OR "automated reasoning" OR "computer vision" OR "smart machine*" OR "deep analytics" OR "big data" OR "decision support system*" OR "data mining" OR "multi-agent systems" OR "health informatics" OR "belief state" OR "online agent" OR "learning agent" OR "strong AI" OR "weak AI" OR "training data" OR "predictive analytics" OR "cognitive automation" OR "intelligent automation" OR "semantic analysis" OR "cognitive computing" OR "AI bias" OR (AI NEAR/3 (judgment OR judgement)) OR (AI NEAR/3 prediction) OR "automation bias" OR "cloud computing" OR deepfake OR "data architecture" OR "computational thinking" OR "general adversarial network*" OR "human machine teaming" OR "human AI teaming" OR "intelligent sensing" OR "object recognition" OR "one shot learning" OR "reinforcement learning" OR "augmented learning" OR "semi-supervised learning" OR explainability OR "generative AI" OR "large language model*") OR MAINSUBJECT.EXACT("Artificial intelligence") OR MAINSUBJECT.EXACT("Expert system programs") OR MAINSUBJECT.EXACT("Man-Machine interfaces") OR MAINSUBJECT.EXACT("Artificial neural network models") OR MAINSUBJECT.EXACT("Robotics") OR MAINSUBJECT.EXACT("Decision support systems") OR MAINSUBJECT.EXACT("Data mining")) AND PEER(yes)  S3  (ti,ab((injur* NEAR/2 preven*) OR (occupation* NEAR/2 (accident* OR disease* OR hazard* OR health OR illness* OR injur* OR safety)) OR (workplace NEAR/2 (hazard* OR injur* OR safety)) OR fatalit*) OR MAINSUBJECT.EXACT("Occupational health and safety") OR MAINSUBJECT.EXACT("Occupational health") OR MAINSUBJECT.EXACT("Occupational diseases") OR MAINSUBJECT.EXACT("Accidents")) AND PEER(yes)  S4  (ti,ab("musculoskeletal disease*" OR "musculoskeletal injur*" OR "carpal tunnel" OR tendonitis OR "neck pain" OR "back pain" OR "shoulder pain" OR osteoarthritis OR "repetitive strain injur*" OR RSI OR "hand-arm vibration syndrome" OR "hand injur*" OR bursitis OR "wounds and injuries" OR "patient risk prevention") OR MAINSUBJECT.EXACT("Musculoskeletal diseases") OR MAINSUBJECT.EXACT("Neck pain") OR MAINSUBJECT.EXACT("Back pain") OR MAINSUBJECT.EXACT("Shoulder pain") OR MAINSUBJECT.EXACT("Osteoarthritis") OR MAINSUBJECT.EXACT("Repetitive strain injuries")) AND PEER(yes)  S5  (ti,ab("psychol* injur*" OR "post-traumatic stress" OR PTSD OR PTSS OR PTSI OR "psychol* trauma" OR technostress OR (psychol* NEAR/2 strain) OR stressor* OR "job strain" OR (job NEAR/2 stress*) OR (work NEAR/2 stress*) OR (career NEAR/2 stress*) OR (employ* NEAR/2 stress*)) OR MAINSUBJECT.EXACT("Psychological trauma") OR MAINSUBJECT.EXACT("Posttraumatic stress disorder") OR MAINSUBJECT.EXACT("Job strain model")) AND PEER(yes)  S6  [S3] OR [S4] OR [S5]  S7  [S1] AND [S2] AND [S6]  S8  ([S1] AND [S2] AND [S6]) AND pd(20180101-20231031) |

**Supplement 2: Quality appraisal criteria and question weight**

| **Systematic review methodological quality criteria** | **Question weight** |
| --- | --- |
| Was there a clear statement of the aims of the research and was the research design appropriate to address the aims of the research? | 4 |
| Were sampling and recruitment methods (including inclusion/exclusion criteria) clearly described and similar for all participants? | 4 |
| Was recruitment (or participation) rate reported and adequate? | 4 |
| Were there important differences between those who participated and did not participate in the study with respect to key characteristics i.e., exposure(s) (including intervention(s)) and outcome(s))? | 4 |
| Were baseline characteristics described? | 4 |
| Was an intervention allocation method performed adequately? | 1 |
| Was the intervention process adequately described to allow for replication? | 4 |
| Was there any potential for contamination? | 4 |
| Was there any potential for co-intervention? | 4 |
| Was compliance with the intervention described and adequate? | 4 |
| Was the length of follow-up 3 months or greater? | 4 |
| Was the loss of follow up (attrition) less than 35%? | 4 |
| Were there important differences between those who completed the study and those who withdrew with respect to key characteristics i.e., exposure(s)? | 4 |
| Were the instruments/methods used to assess exposure(s) valid and reliable? | 4 |
| Were the instruments/methods used to assess the outcome(s) valid, reliable, and not prone to important sources of measurement bias? | 6 |
| Were the outcomes described at baseline and follow-up? | 6 |
| Was data collection with respect to exposure/outcome carried out equivalently for all participants? | 6 |
| Were all participants’ outcomes analyzed by the groups to which they were originally allocated (intention-to-treat analysis)? | 6 |
| Were important covariates, confounders, or baseline differences (if necessary) accounted for in the study design and/or analysis? | 6 |
| Was there a direct between group comparison? | 6 |
| Was the design of the AI described? | 4 |
| Was the data, which was used to train the algorithm, described? | 4 |
